# The G-protein coupled receptor 56, expressed in colonic stem and cancer cells, binds progastrin to promote proliferation and carcinogenesis

**DOI:** 10.18632/oncotarget.16506

**Published:** 2017-03-23

**Authors:** Guangchun Jin, Kosuke Sakitani, Hongshan Wang, Ying Jin, Alexander Dubeykovskiy, Daniel L. Worthley, Yagnesh Tailor, Timothy C. Wang

**Affiliations:** ^1^ Division of Digestive and Liver Diseases, Department of Medicine, Columbia University Medical Center, New York, NY, USA; ^2^ The Research Institute, Yanbian University Hospital, Jilin, China; ^3^ Department of General surgery, Zhongshan Hospital, Fudan University, Shanghai, China; ^4^ South Australian Health and Medical Research Institute, University of Adelaide, Adelaide, South Australia, Australia

**Keywords:** GPR56, progastrin, proliferation, stem cell, colorectal cancer

## Abstract

Overexpression of human progastrin increases colonic mucosal proliferation and colorectal cancer progression in mice. The G-protein coupled receptor 56 (GPR56) is known to regulate cell adhesion, migration, proliferation and stem cell biology, but its expression in the gut has not been studied. We hypothesized that the promotion of colorectal cancer by progastrin may be mediated in part through GPR56. Here, we found that GPR56 expresses in rare colonic crypt cells that lineage trace colonic glands consistent with GPR56 marking long-lived colonic stem-progenitor cells. GPR56 was upregulated in transgenic mice overexpressing human progastrin. While recombinant human progastrin promoted the growth and survival of wild-type colonic organoids *in vitro*, colonic organoids cultured from GPR56^−/−^ mice were resistant to progastrin. We found that progastrin directly bound to, and increased the proliferation of, GPR56-expressing colon cancer cells *in vitro*, and proliferation was increased in cells that expressed both GPR56 and the cholecystokinin-2 receptor (CCK2R). *In vivo*, deletion of GPR56 in the mouse germline abrogated progastrin-dependent colonic mucosal proliferation and increased apoptosis. Loss of GPR56 also inhibited progastrin-dependent colonic crypt fission and colorectal carcinogenesis in the azoxymethane (AOM) mouse model of colorectal cancer. Overall, we found that progastrin binds to GPR56 expressing colonic stem cells, which in turn promotes their expansion, and that this GPR56-dependent pathway is an important driver and potential new target in colorectal carcinogenesis.

## INTRODUCTION

Colorectal cancer (CRC) is the third most commonly diagnosed cancer in the world [1]. CRC develops through a series of somatic gene mutations that promote malignant behavior [2]. These traditional pathway mutations, propagated by long-lived progenitor cells, lead to activation of proliferative signaling pathways, crypt fission, and aberrant glands that progress pathologically from aberrant crypt foci into small adenomatous polyps and ultimately invasive adenocarcinoma. Hyperproliferation of colonic mucosal epithelial cells has been found to be a critical initiating event in colorectal carcinogenesis [3]. Growth factors and hormones can modulate colonic mucosal epithelial cells proliferation and thus an individual's underlying predisposition to colorectal cancer.

Preprogastrin is synthesized by gastric G cells, and cleavage of the signal peptide at the C-terminus leads to the production of progastrin [4]. Progastrin can undergo further processing to generate glycine-extended and amidated forms of gastrin. While progastrin and other nonamidated gastrins typically comprise less than 10% of the total secreted peptide, elevated levels have been described in some gastrointestinal cancer patients [4, 5], and progastrin has been shown to be expressed by numerous primary human tumors and cancer cell lines [6–8]. Amidated gastrins were initially thought to be the only biologically active form of gastrin. However, in recent years, progastrin has been recognized as a growth factor. Overexpression of human progastrin in transgenic mice, or progastrin treatment *in vitro*, stimulates colonic epithelial proliferation and colonic carcinogenesis, proving that progastrin is a trophic growth factor for colonic epithelium in mice [9–12]. Furthermore, overexpression or exogenous administration of progastrin increases colonic epithelial proliferation and regeneration after DNA damage by gamma-radiation or chemical carcinogens [13]. These effects of progastrin were independent of other forms of amidated gastrin [14].

Recent studies have suggested that progastrin's effects on colonic mucosal proliferation and carcinogenesis are mediated through inhibition of bone morphogenetic protein (BMP) signaling [11]. Overall, while proliferative and protumorigenic effects of progastrin have been demonstrated both *in vivo* and *in vitro*, the receptors and pathways that mediate progastrin induced proliferation remain somewhat controversial. Several high affinity binding proteins of progastrin have been reported by other groups [11, 15–18]. Recent reports by our group have demonstrated that the cholecystokinin type 2 receptor (CCK2R) when expressed in colon cancer cells can bind to progastrin, while antagonism or knockout of the CCK2R receptor *in vivo* abrogates progastrin's proliferative and protumorigenic effects [11]. CCK2R is expressed, however, only at very low levels in the normal murine colon, thus raising the question as to whether CCK2R is indeed the primary receptor mediating progastrin's effects.

As an orphan G protein–coupled receptor (GPCR), GPR56, is a member of the class secretin-like GPCR subfamily with an extremely long extracellular domain thought to play a role in cell-cell and cell-matrix interactions [19]. GPR56 is highly expressed in the brain, thyroid gland and heart, with moderate levels in kidney and pancreas, small intestine, stomach, and colon [19, 20]. In the brain, GPR56 is expressed in the germinal zones of fetal and adult brain regions harboring neural stem cells, and there is a strong link between GPR56 and stem cell function across a wide range of distinct compartments. For instance, deficiency of GPR56 gene expression impairs neurogenesis, while overexpression increases proliferation and progenitor number in neuron [21]. Mutations in GPR56 have been linked to bilateral frontoparietal polymicogyria [22], which is due to altered migration and proliferation of neuronal stem cells during brain development [23]. GPR56 has also been shown by Irving Weissman's group to be expressed in hematopoietic stem cells [24]. Taken together, these data raise the possibility that GPR56 may function to control the proliferation or behavior of multipotent stem cells of diverse origins. GPR56 does not appear to be required for survival of adult mammals since knockout mice are viable [25]. Although GPR56 may also interact with tissue collagen III and transglutaminase 2 [26, 27], specific ligands have not been identified and GPR56 has remained classified as an “orphan receptor” with unknown functions.

In addition, GPR56 is overexpressed in numerous cancers, including glioblastomas, breast, pancreatic, renal, esophageal cancers, and colon cancer [20, 28–30]. In some studies, significantly elevated levels of GPR56 were observed in transformed cells compared with its isogenic nontransformed revertant, and GPR56 silencing by RNAi approaches led to growth suppression *in vitro* and tumor regression in xenograft tumor models *in vivo* [28]. A smaller number studies have pointed to a possible role for GPR56 as a tumor suppressor gene as it is downregulated in the setting of metastasis [26], suggesting tissue specific effects in cancer. GPR56 has been shown to interact with both Gaq/11 and Gq12/13, and activate a number of downstream signaling pathways including ERKs, NF-kB, cAMP, and most importantly Wnt signaling [31, 32]. Studies by Shashidhar et al have shown that GPR56 overexpression results in the upregulation of TCF reporter genes, implicating the beta-catenin pathway in GPR56 signaling [30].

In this study, we demonstrated that progastrin binds to GPR56- expressing colon cancer cells, and utilizing GPR56-CreER™ transgenic mice, that GPR56 is expressed in a subset of stem cells in the colonic crypt. Deletion of GPR56 abrogates progastrin-dependent colonic crypt fission, proliferation and colorectal carcinogenesis in mice. Although a few GPCRs have been considered as potential cancer drug targets, our studies suggest that GPR56 plays an important role in mediating the effects of progastrin induce colonic proliferation and colon carcinogenesis and thus could serve as a valuable future target to prevent and treat colorectal carcinogenesis.

## RESULTS

### GPR56 is expressed in murine colonic crypt cells and upregulated in human progastrin transgenic mice

**Figure 1:**
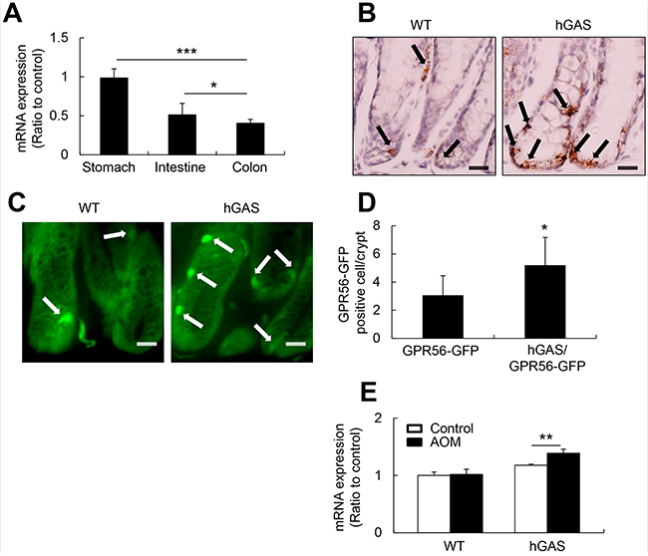
GPR56 expresses in the murine colonic mucosa and upregulates in the hGAS mice colon **(A)** Quantitative RT-PCR analysis of GPR56 mRNA expression levels in WT mouse stomach, small intestine, and colon (*n* = 4/group). mRNA was prepared, cDNA was synthesized, and qRT-PCR was performed. **(B)** In situ hybridization to detect murine GPR56 mRNA with double “Z” oligo probes in the WT and hGAS mouse colonic mucosa. Scale bar: 20 μm (original magnification, ×600). **(C)** GPR56-EGFP expression in the colonic mucosa of WT and hGAS mice. Scale bar: 20 μm (original magnification, x600). **(D)** Quantification of GPR56-EGFP positive cells in the colonic mucosa of WT and hGAS mice (*n* = 4 mice/group). **(E)** qRT-PCR analysis of GPR56 mRNA expression in each group of mice colonic mucosa (*n* = 4 mice/group). The expression of GPR56 mRNA in the colon was analyzed two weeks after biweekly injections of AOM (10 mg/kg). The expression levels were normalized to GAPDH. All values represent the mean ± SD. *P < 0.05, **P < 0.01, ***P < 0.001.

While GPR56 is widely expressed in murine neuronal, muscle, and thyroid cells [19, 33], the expression of GPR56 in the gastrointestinal epithelium has not been defined. Using quantitative RT-PCR (qRT-PCR) analysis, we confirmed that mRNA expression level of GPR56 was higher in the stomach than in the small intestine and colon in 6-week-old WT C57BL/6 mice (Figure 1A). Additionally, in situ hybridization of GPR56 (Figure 1B) and immunofluorescence analysis of GPR56-EGFP (Figure 1C) detected GPR56 positive epithelial cells located near the base of the colonic crypts. In addition, more numerous GPR56-expressing cells could be detected in progastrin-overexpressing hGAS/GPR56-EGFP mice compared to the WT/GPR56-EGFP mice (Figure 1D). Furthermore, the carcinogen AOM induced a significant increase the mRNA expression levels of GPR56 in hGAS mice colonic mucosa compared to the WT mice (Figure 1E). Taken together, these observations suggest that increased progastrin expression in hGAS mice leads to increases in GPR56-expressing cells, particularly in the setting of carcinogenic injury.

### GPR56 identifies bone fide colonic progenitor cells

**Figure 2:**
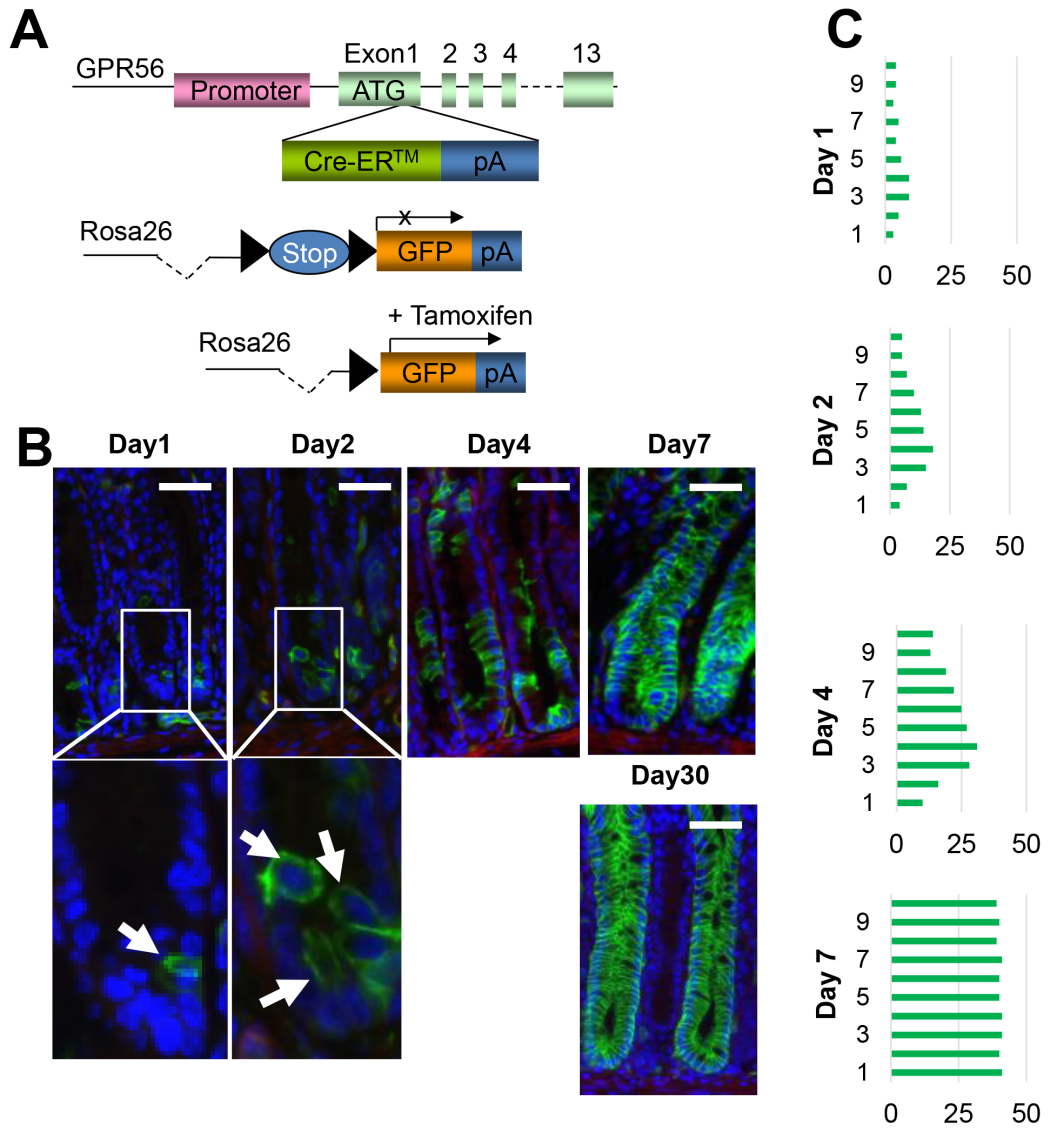
GPR56 labels colonic stem or progenitor cells **(A)** Schematic diagram of GPR56-CreER™ recombinase responsive allele. The expressing encoding sequence is activated by the depletion of loxP-flanked STOP cassette. **(B)** Immunofluorescences of GPR56 (green) in GPR56-CreER™/ROSA26r-EGFP mice (after 3 mg tamoxifen induction at day 1, 2, 4, 7 and 30). Scale bar: 50 μm. **(C)** Quantification of the GPR56-traced cell position. The total 50 glands are analyzed at each time point.

In order to determine the nature of GPR56-expressing cells, and whether they might represent colonic stem or progenitor cells, we used BAC recombineering to generate GPR56-BAC-CreER™ transgenic mice (Figure 2A). Several lines of mice were produced and crossed to ROSA26r-EGFP reporter mice. At one day following Tamoxifen induction, the mice showed expression in rare colonic crypt cells, similar to the GPR56-EGFP reporter mice. Treatment with Tamoxifen resulted in traced colonic crypt cells that expanded over time, and by 7 days this resulted in complete lineage tracing of the majority of the colon glandular units (Figure 2B, 2C). These colonic glands remained labeled for more than 60 days (not shown). Thus, GPR56 identifies long-lived colonic progenitor cells.

### Progastrin increases colorectal cancer cell proliferation through GPR56

**Figure 3:**
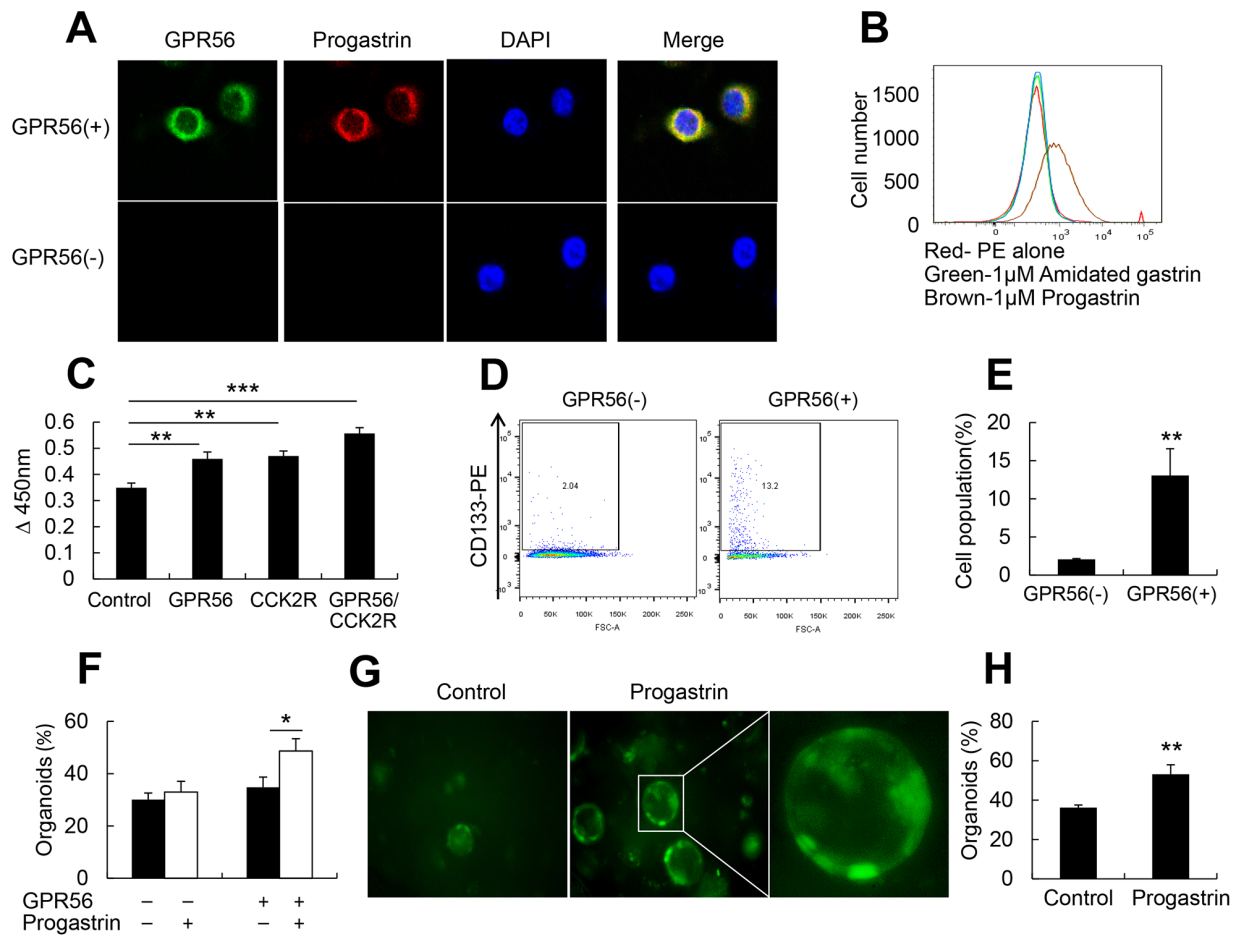
Progastrin binds to GPR56 to increase cell proliferation **(A)** Immunofluorescence staining of progastrin binds on GPR56-expressing cells after 30 minutes of progastrin treatment (original magnification, x800). **(B)** FACS analysis after 30 minutes of progastrin and amidated gastrin binding to the GPR56(+) cells. **(C)** MTT assay after progastrin treatment on GPR56, CCK2R, and GPR56/CCK2R expressing cells. **(D)** FACS analysis of CD133 positive colon cancer cells after two days culture in progastrin. **(E)** Quantification of CD133 positive cell populations after progastrin treatment to the GPR56(-) and GPR56(+) cells (n=3 plates/group). **(F)** The percentage of survived colonic crypt organoids from WT and GPR56^−/−^ mice (n=3 plates/group) after 3 days in culture with or without progastrin (1 nmol/ml). **(G)** Representative pictures of colonic organoids from GPR56-EGFP mice (original magnification x200). **(H)** The percentage of GFP positive colonic crypt organoids from GPR56-EGFP mice after three days of progastrin treatment. All values represent the mean ± SD. *P < 0.05, **P < 0.01, ***P < 0.001.

With qRT-PCR analysis of GPR56 mRNA expression, GPR56-EGFP mice and GPR56-CreER™ mice study, we confirmed that GPR56 is expressed in progenitor cells in the mice colonic crypts. To investigate whether progastrin binds to GPR56 and regulates colonic epithelium cell proliferation, we stably transfected the colorectal cancer cell line (Colo320) with an expression construct for GPR56. After treating the cells with 1 nmol/ml recombined human progastrin (1-80) for 30 minutes, we found that progastrin binds to the surface of GPR56-expressing cells but not to non-expressing control cells (Figure 3A). Furthermore, through fluorescence activated cell sorting (FACS), we found that progastrin binds to the GPR56 (+) colon cancer cell surface, while amidated gastrin (G-17) did not (Figure 3B). To investigate whether progastrin increases cell proliferation through GPR56, we treated the GPR56-expressing cells with progastrin in serum free medium. Doses response studies (not shown) showed significant responses with 0.5 nmol/ml and maximal responses with 1 nmol/ml of progastrin. After 48 hours with 1 nmol/ml of progastrin, we found significantly increased cell proliferation, and this effect was augmented in the GPR56/CCK2R double positive cells (Figure 3C). Next, we treated the GPR56 positive colon cancer cells for two days with 1 nmol/ml of progastrin, FACS analyzed the CD133 positive cell population, and found significantly increased CD133 expression in GPR56 expressing cells (Figure 3D, 3E). Overall, these results suggest that progastrin not only binds to the GPR56 but increases GPR56 positive cell proliferation leading to the conclusion that the expression of GPR56 is both necessary for progastrin binding and proliferation in colonic epithelial cells.

Colonic organoid culture can also be used to directly investigate *in vitro* progenitor function in normal colonic crypts [34]. To determine whether progastrin regulates colonic epithelial cells proliferation through GPR56, we isolated colonic crypts from GPR56^−/−^, WT, and GPR56-EGFP mice. With progastrin treatment (1 nmol/ml) for 3 days in culture, we found an increase in survival in WT colonic organoids compared to GPR56^−/−^ mice (Figure 3F) with prolonged GPR56-EGFP expression (Figure 3G, 3H), suggesting that progastrin mediated colonic epithelium proliferation effects were largely dependent on GPR56.

### Deletion of the GPR56 gene inhibits progastrin-dependent colonic mucosa proliferation and increases colonic apoptosis

**Figure 4:**
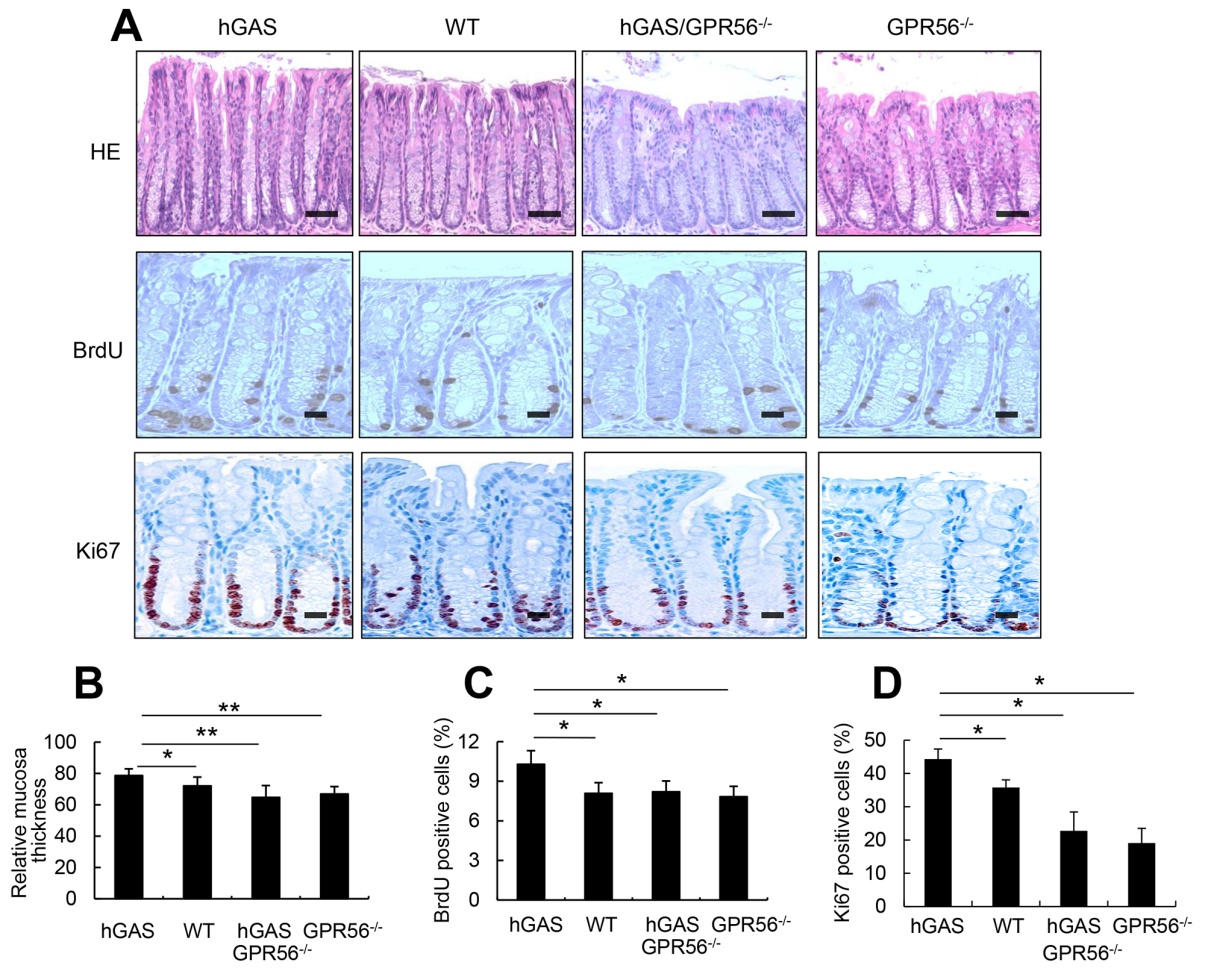
Inactivation of the GPR56 gene inhibits progastrin-dependent colonic proliferation **(A)** Top: Hematoxylin and eosin–stained sections from the distal colon show mucosa thickness in each group of mice (original magnification, x200). Scale bar: 100 μm. Middle: Immunohistochemical staining of BrdU positive cells in the distal colon from each group of mice after intraperitoneal injection of BrdU (original magnification, x400). Scale bar: 25 μm. Bottom: Immunohistochemical staining of Ki67 positive cells in the colon from each group of mice (original magnification, x400). Scale bar: 25 μm. **(B)** Relative colonic mucosal thickness was measured at three different locations (proximal, middle, and distal colon) in mice from 4 groups (n = 4/group). Mucosa thickness was measured with Nikon TE2000 microscope image analysis software. The percentages of BrdU-positive cells **(C)** and Ki67-positive cells **(D)** in the colonic crypts were measured at 30 crypts in different locations in mice colons from each group (n = 4/group). All values represent the mean ± SD. *P < 0.05, **P < 0.01.

In order to confirm *in vivo* a role for GPR56 in progastrin-dependent colonic proliferation, we crossed human progastrin transgenic mice (hGAS) with GPR56-deficient mice. We generated four groups of mice on a C57BL/6 background: hGAS, WT, hGAS/GPR56^−/−^, and GPR56^−/−^. Histopathologic analysis revealed an increased number of cells per crypt, and increased colonic crypt height, in colons of hGAS mice compared to the other three groups (WT, hGAS/GPR56^−/−^, GPR56^−/−^) (Figure 4A). Colonic mucosal crypt thickness at three different locations (proximal, middle, and distal colon) was significantly higher in the hGAS mice colon compared to the other three groups of mice (Figure 4B). Colonic proliferation as measured using BrdU incorporation and Ki67 immunostaining confirmed that the majority of proliferating cells were located in the lower third of the colonic crypts (Figure 4A). Furthermore, quantification of BrdU+ and Ki67+ cells, relative to total epithelial cell number in 30 randomly chosen crypts, showed significantly increased proliferation in hGAS mice compared to the other three groups of mice (WT, hGAS/GPR56^−/−^, GPR56^−/−^) (Figure 4C, 4D).

**Figure 5:**
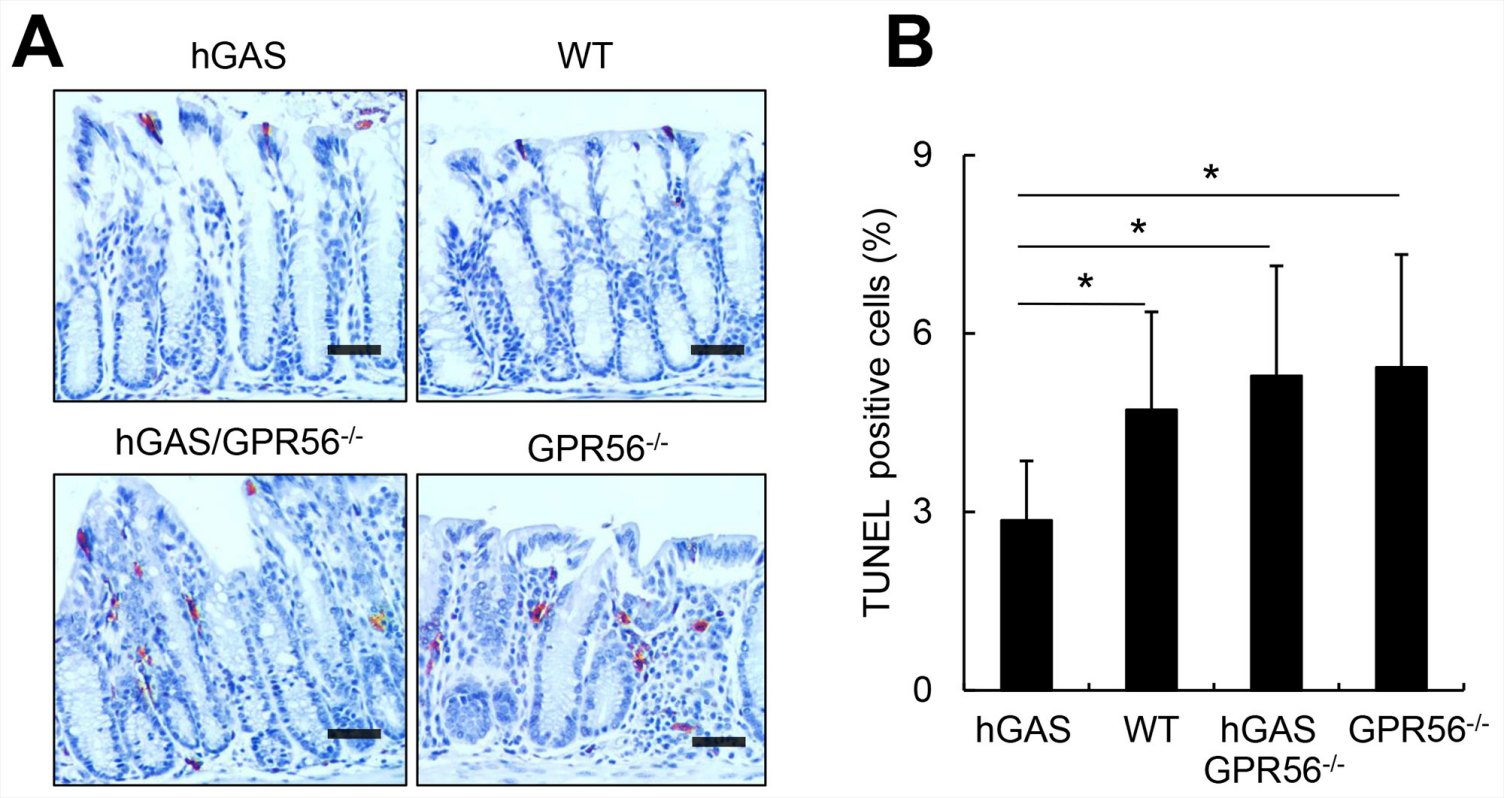
Inactivation of the GPR56 gene increases colonic apoptosis in hGAS mice **(A)** Immunohistochemical staining of TUNEL-positive cells in the colon from each group of mice (original magnification, x200). Representative results from 4 different groups are shown. Scale bar: 50 μm. **(B)** Percentage of TUNEL-positive cells in different locations in mice colons from each group (n = 4/group). All values represent the mean ± SD. *P < 0.05.

In addition, the distribution of apoptotic cells in the colonic crypts as revealed by TUNEL assay was markedly different in all groups. Interestingly, the hGAS mice showed the fewest apoptotic cells, with most of them found on the mucosal surface (Figure 5A). Quantitative analysis of TUNEL+ cells throughout the colonic crypts showed significant differences, with fewer apoptotic colonic cells in hGAS mice compared to the WT, hGAS/GPR56^−/−^ and GPR56^−/−^ mice (Figure 5B). Thus, these results suggest that deletion of the GPR56 gene inhibited colonic epithelial proliferation in progastrin-overexpressing mice but not in WT mice. Moreover, knockout of GPR56 was sufficient to block the progastrin-dependent inhibition of colonic epithelial apoptosis.

### Inactivation of the GPR56 gene inhibits progastrin-dependent colonic ACF and colorectal cancer generation

**Figure 6:**
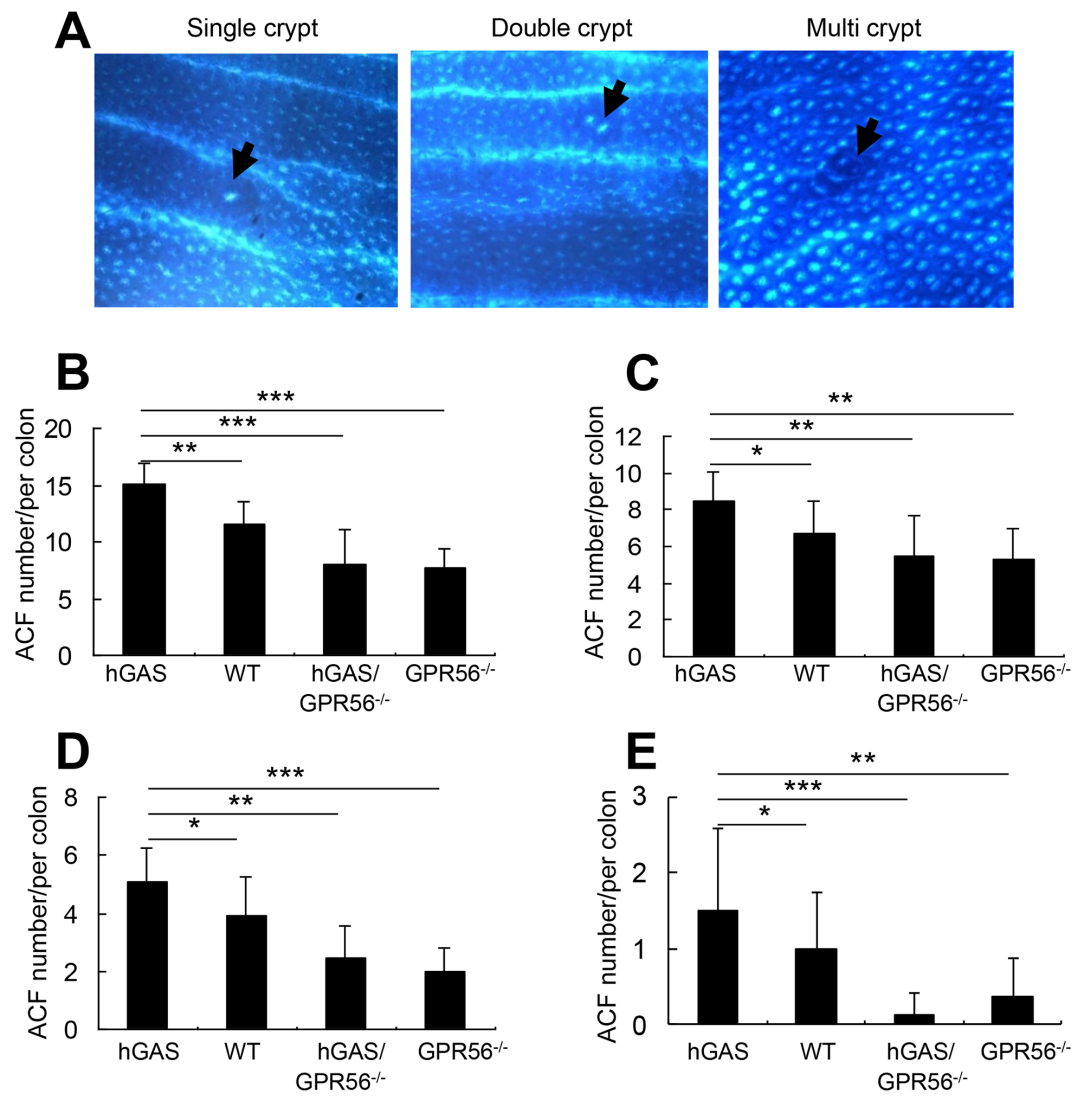
Inactivation of the GPR56 gene inhibits progastrin-dependent colonic ACF formation **(A)** Representative pictures of the three different types of ACF (single, double, and multiple crypt) in the colon of AOM-treated mice (original magnification, x200). The arrows indicate the location of the ACFs. Colons were removed at two weeks after biweekly injections of AOM and fixed with 70% ethanol overnight. The ACFs were analyzed under a light microscope after methylene blue staining. Total number of ACFs **(B)** and the number of single ACF **(C)**, double ACF **(D)**, and multiple ACF **(E)** in each colon of mice (n = 4/group). All values represent the mean ± SD. *P < 0.05, **P < 0.01, ***P < 0.001.

Aberrant crypt foci (ACF) are clusters of abnormal tube-like glands in the colon and rectum. ACFs form prior to the formation colonic polyps, and are thought to be one of the earliest detectable precursor lesions for colorectal cancer [35]. In order to investigate the role of GPR56 deficiency in the formation of ACFs, AOM was given once a week for two weeks to all 4 groups of mice. Two weeks after the last injection, ACFs were found in all groups of AOM-treated mice, with most of the ACFs present in the distal half of the colon. ACFs were classified according to multiplicity into three types: single crypt, double crypt, and multi-crypt ACFs (Figure 6A). Overall, hGAS mice had significantly more ACFs of all 3 types (Figure 6B), single crypt (Figure 6C), double crypt (Figure 6D), and multi-crypt (Figure 6E) compared to the other 3 groups of mice. The greatest difference was apparent for multi-crypt ACFs, which were abundant in hGAS mice but completely suppressed in hGAS/GPR56*^–/–^* mice (Figure 6E).

**Figure 7:**
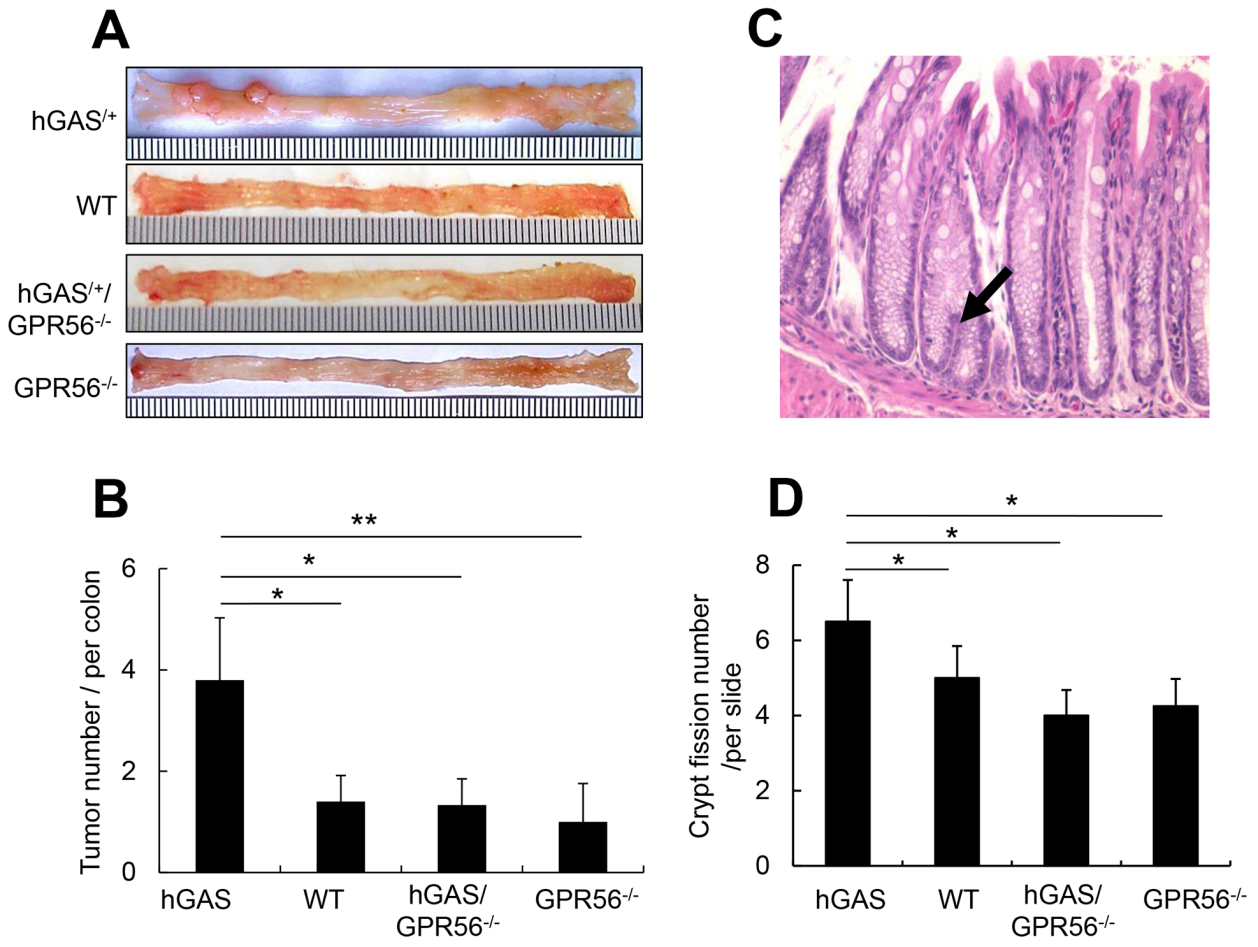
Deletion of the GPR56 gene inhibits progastrin-dependent colorectal tumor formation and colonic crypt fission **(A)** Macroscopic changes and tumor formation in colons of mice from each of 4 groups. Colons were removed at 18 weeks after one time of AOM injection (i.p) followed by one week of 3% DSS treatment in drinking water. Representative results from 4 independent animals are shown. **(B)** Total number of visible tumors (n = 4/group). **(C)** Representative picture of 6-week old mouse distal colonic mucosa with crypt fission (arrows) (original magnification, ×300). **(D)** Relative number of mouse colonic crypt fissions per section of slide (n = 4/group). All values represent the mean ± SD. *P < 0.05, **P < 0.01.

Additionally, the four groups of mice were given one dose of AOM followed by one week of 3% DSS treatment, resulting in the development of colonic tumors in all four groups of mice at 18 weeks. Similar to the localization of ACFs, tumors in all four groups of mice were located primarily in the distal half of the colon, with only a few tumors present in the proximal half of the colon (Figure 7A). Overall, the tumors were more frequent and much larger in the hGAS mice. The average tumor number in hGAS mice was significantly higher than in WT, hGAS/GPR56^–/–^, and GPR56^–/–^ mice (Figure 7B). Moreover, GPR56 deficiency markedly suppressed the growth of large (> 3 mm) tumors, as there were no large tumors present in WT, hGAS/GPR56^–/–^, and GPR56^–/–^ mice (Figure 7A).

### Progastrin overexpression increases colonic progenitor cells expansion and crypt fission through GPR56

Colonic crypt fission is a physiologic mechanism needed for colonic mucosa regeneration. Typically, increased crypt fission requires symmetric division of colonic epithelial stem cells located near the bottom of the crypts [36]. The expansion of colonic stem cells is thought to promote colonic growth in part through crypt fission, such that intestinal crypts divide in response to a doubling of stem cell number [37]. In this study, we found a significant increase in crypt fission in hGAS mice (Figure 7C) predominantly in the distal colon. In contrast, we observed a much lower incidence of crypt fission in WT, hGAS/GPR56^−/−^, and GPR56^−/−^ mice (Figure 7D).

## DISCUSSION

Progastrin is an established colonic growth factor, able to increase mucosa proliferation and promote carcinogenesis of the human and mouse colon [7, 9, 12]. In the current study, we found that progastrin mediates its colonic proliferation and tumorigenesis, at least in part, via the G-protein coupled receptor 56 (GPR56). We show that GPR56 is expressed in murine colonic crypt epithelial cells, and using inducible Cre-ER™ lineage tracing, demonstrate that these cells are bone fide colonic stem cells. Inactivation of GPR56 *in vivo* inhibited progastrin-induced colonic proliferation, and increased colonic epithelial apoptosis. This colonic stimulatory effect by progastrin in hGAS mice was associated with expansion of GPR56 positive cells, which was enhanced after azoxymethane (AOM) treatment. *In vitro*, progastrin increased the growth and survival of colonic organoids, while deletion of GPR56 abrogated this effect. Finally, progastrin overexpression increased AOM-dependent aberrant crypt foci (ACF) and tumorigenesis in the mouse colon, and the effects were blocked by GPR56 deletion.

Although progastrin has been shown in many studies to stimulate colonic proliferation and tumorigenesis, the mechanisms involved are not well understood [10, 38]. Annexin II has been shown to bind progastrin and mediate some of progastrin's proliferative effects, but to date has not been shown to be expressed specifically in the colonic crypts [38]. The CCK2 receptor also bind progastrin and is expressed at low levels in colonic crypts [10], but studies from other labs have indicated that some of progastrin's proliferative effects are likely to be independent of CCK2R [39]. GPR56, a member of the G protein-coupled receptor family, has been shown to regulate oligodendrocyte progenitor cell proliferation [40]. Here, we show that progastrin was able to bind to GPR56 and stimulate colon cancer cell proliferation. Progastrin-overexpressing hGAS mice exhibited upregulation of both GPR56 mRNA and protein expression in the colon. Using both GPR56-EGFP and a novel GPR56-CreER™ transgenic line, we show that GPR56 is expressed in rare colonic crypt cells that have characteristics of colonic stem cells. Tamoxifen-induced Cre recombination in GPR56-CreER™/Rosa26-TdTomato mice led to lineage tracing of entire colonic glands. Studies using GPR56^−/−^ mice show clearly that GPR56 is required for progastrin-dependent colonic proliferation and tumorigenesis in mice.

Furthermore, in addition to modulating colonic proliferation, progastrin acts through GPR56 to promote colorectal cancer progression. AOM-dependent ACFs and colonic tumors were significantly decreased in the hGAS/GPR56^−/−^ mice compared to hGAS mice, and knockout of the GPR56 gene markedly reduced the multiplicity of ACFs and large colon tumors. ACFs, enlarged aberrant elliptical shaped crypts, could easily be differentiated from normal crypts and are considered to be early lesions in colon carcinogenesis [41]. Thus, our findings suggest that the progastrin/GPR56 can act very early in the colorectal cancer pathway.

Given our previous studies demonstrating a role for the CCK2R in progastrin's proliferative effects in the colon, questions arise as to the relative importance of this receptor compared to GPR56. Knockout of either gene appears to inhibit progastrin-induced proliferation and colonic tumorigenesis. In the case of GPR56, gene knockout suppressed baseline BrdU- and Ki67- positive colonic epithelial cells, suggesting the presence of endogenous signaling, or stimulation by low levels of circulating progastrin. Both CCK2R and GPR56 are G-protein-coupled receptors, and co-expression in cells led to further increases in cell proliferation after progastrin treatment. However, further study is needed to determine whether there is cross-talk between receptors, or whether other mechanisms (e.g. heterodimerization) may be operational.

The functional importance of GPR56 in colonic mucosa proliferation and colon cancer generation appear to be related to its localization in the crypts in colon stem and progenitor cells. Previous studies have shown that recombinant human progastrin bound competitively to the colonic crypts [42]. In this study, we were able to localize GPR56 positive cells to the colonic crypts in GPR56-EGFP mice, and found that the number of GPR56 cells was increased in mice that overexpressed progastrin. We also have demonstrated through lineage tracing that GPR56 marks bone fide colonic stem cells. Furthermore, we found that progastrin treatment of GPR56 expressing cells lead to increased CD133 positive cells. In the setting of colon carcinogenesis, progastrin stimulated proliferation and colonic crypt fission in a GPR56-dependent manner. Crypt fission is a physiologic phenomenon of crypt generation most commonly found in neonatal animals and humans that require rapid colonic mucosal growth [43], but commonly found in colonic regeneration and preneoplasia, and is thought to be associated with stem cell expansion [44, 45]. Taken together, these results strongly suggest that progastrin stimulates proliferation and tumorigenesis by stimulation of GPR56-expressing colonic stem and progenitor cells. Future studies are needed to explore the utility of targeting GPR56 in cancer prevention and therapy.

## MATERIALS AND METHODS

### Animals

hGAS transgenic mice on an C57BL/6 background were crossed with GPR56^−/−^ mice (F9 backcross to C57BL/6 strain). Breeding resulted in littermates that included mice of hGAS, hGAS/GPR56^−/−^, GPR56^−/−^, and WT genotypes. GPR56^−/−^ mice were provided by Dr. Lei Xu (University of Rochester, New York, USA). GPR56-EGFP mice were obtained from The Mutant Mouse Regional Resource Center (MMRRC). Gt(ROSA)26Sortm4(ACTB-tdTomato,-EGFP)Luo/J mice were purchased from Jackson Laboratories. GPR56-CreER™ mice were generated by the insertion of CreER™-Frt-Neo-Frt cassette after ATG start codon in exon 1 as described previously [46]. All mice were bred and maintained under specific pathogen–free conditions at the animal facility of Columbia University Medical Center (CUMC). All experiments were approved by the Subcommittee on Institutional Animal Care and Use at Columbia University Medical Center.

In the study of mouse colonic mucosa proliferation, four 6-week-old male mice in each group were injected intraperitoneally with 50 mg/kg BrdU (Sigma-Aldrich) 1 hour before being sacrificed. To induce ACFs, 4 groups of 6-week-old sex-matched mice (*n* = 12/group) were given weekly intraperitoneal injections of 10 mg/kg azoxymethane (Sigma-Aldrich) in PBS for 2 weeks. Two weeks after the last injection, the mice were sacrificed and full-length colons were removed. Longitudinally dissected colons were spread on Whatman filter paper, followed by fixation with 70% ethanol for 24 hours. After staining with 0.3% methyl-blue for 1 minute, the total number of ACFs per colon was counted under a light microscope. To induce colorectal cancer, 4 groups of 6-week-old mice (*n* = 4/group) were injected one time of AOM (10 mg/kg) followed by one week of 3% DSS in drink water. Eighteen weeks after AOM injection, the mice were sacrificed, colons were removed, and the total number of tumors per colon was analyzed.

### Histopathological and immunohistochemical analysis

Resected murine colons were rolled onto a plastic bar using the Swiss roll technique [47], and fixed in 10% formalin (VWR International) for paraffin embedding or fixed in 4% paraformaldehyde for freezing in Tissue-Tek O.C.T. compound (Sakura). Paraffin-embedded tissues were cut in 4-μm slices and deparaffinized for hematoxylin and eosin staining and additional immunohistochemical analysis. The deparaffinized colon tissues were blocked with peroxidase blocking solution for 5 minutes, then with 2% bovine serum albumin (Sigma-Aldrich) for 1 hour, followed by incubation in anti-BrdU and anti-Ki67 (Abcam Inc) antibodies for 1 hour. Samples were washed and incubated with HRP-conjugated anti-rat or anti-rabbit IgG (Dako). The epithelial cell proliferation rate was expressed as the number of BrdU- or Ki67 positive cells divided by the total number of cells in each crypt. Thirty crypts in different sites of each colon were randomly chosen. For analysis of GPR56-EGFP cells expression in the colonic crypt, the frozen slides were washed three times with PBS and mounted with mounting media with DAPI (DAKO), and analyzed under a Nikon TE2000 microscope.

### Gene expression analyses

For qRT-PCR analysis, colons from 6-week-old male mice were homogenized with IKA ULTRA-TURRAX Dispersers (IKA Works Inc.). The RNA was isolated using the TRIzol (Invitrogen) reagent, and reverse transcription was performed using the Super Script III First-Strand Synthesis System (Invitrogen). qRT-PCR was performed on a 7300 Real-Time PCR System (Applied Biosystems) using the comparative Ct quantitation method with QuantiTect SYBR Green PCR kit. Reactions were done at 95°C for 15 minutes, followed by 40 cycles of 94°C for 15 seconds, 55°C for 20 seconds, and 72°C 30 seconds.

### Cell lines and cell culture

The human colorectal cancer cell line Colo320 expressing CCK2R was a kind gift of Dr. Graham Baldwin. The GPR56 expressing cell line Colo320 (ATCC) was generated by transfection with a plasmid expression construct containing the cDNA encoding the human GPR56 (Addgene, Cambridge, MA). GPR56-positive cells were selected with 5 μg/ml puromycin (Fisher Scientific) treatment for 2 weeks. Afterward, the cells were maintained in F-12K medium containing with 3 μg/ml puromycin.

### Cell proliferation assays

Cells (1×10^4^) were plated in 96-well plates 200 ul F-12K medium containing 10% FBS. After 24 hours, the medium was replaced with serum-free medium and the cells were cultured additional 24 hours, and then the cells were treated with 1 nmol/ml human progastrin (New England Peptide, Gardner, MA). After 48 hours, the cell viability was assessed using an MTT assay.

### Progastrin binding assay

GPR56-expressing Colo320 cells were treated with 1 nmol/ml recombinant human progastrin for 30 minutes. Afterward, the cells were washed three times with PBS and fixed with 4% paraformaldehyde. After incubation with 2% BSA for 1 hour to block unspecific binding, the cells were incubated 30 minutes in prepared anti-progastrin antibody (Santa Cruz Biotechnology) and anti-GPR56 antibody (Santa Cruz Biotechnology). Cells were then washed and incubated with Alexa Fluor 594 anti-goat IgG (Invitrogen) and Alexa Fluor 488 anti-rabbit IgG (Invitrogen), and analyzed under a Nikon TE2000 microscope.

### Colonic crypt isolation and culture

The isolation and culture of murine colonic crypts was performed according to previous published protocols [48]. Mice were euthanized with isoflurane, and colons opened and washed with cold phosphate-buffered saline (PBS). Specimens were cut into 5-mm pieces and then incubated in 8 mM EDTA for 60 minutes on ice followed by the vigorous suspension with a 10-mL pipette. Colonic tissues were centrifuged at 900 rpm for 5 min and diluted with Dulbecco's modified Eagle's medium/F-12 (Invitrogen) containing B27, N2, 1 mM n-acetylcysteine, 10 mM HEPES, penicillin/streptomycin, and Glutamax (Invitrogen). After passing through a 100-mm cell strainer (BD Biosciences), the tissues were centrifuged at 720 rpm for 5 minutes and the single cells were removed. The isolated crypts were embedded equally in 24 well plates (300 crypts/well) in Englebroth-Holm-Swarm sarcoma matrix (Bethesda). After solidification of the matrix, conditional Dulbecco's modified Eagle's medium/F-12 medium containing 1 mg/mL R-spondin1, 50 ng/mL epidermal growth factor (Invitrogen), 100 ng/mL Noggin (Peprotech), 50 ng/mL gentamycin (Sigma), and Wnt3a were overlaid. Growth factors were added every other day, and progastrin was added to the cell culture mediums every day. The numbers of colonic organoids were counted using standard microscopy, and the images were acquired under the fluorescence microscopy to assess EGFP expression. The percentage of surviving organoids after three days in culture was calculated as the number of viable organoids per well divided by the number of crypts plated at the start.

### Flow cytometry analysis and fluorescence activated cell sorting

4 x10^4^ Colo320 cells were plated in 6-well plates with F-12K medium containing 10% FBS. After 24 hours, the cells were replaced with serum-free medium and cultured for an additional 24 hours. The cells were then treated with 1 nmol/ml human progastrin in serum-free medium. After 48 hours, the cells were harvested and incubated for 30 minutes at room temperature with the antibody of PE conjugated rat anti-CD133 (BioLegend), and analyzed using a fluorescence-activated cell sorter LSRII flow cytometer (Becton Dickinson, San Jose, CA). To the progastrin binding assay, the cells were treated with 1 nmol/ml biotinylated amidated gastrin or 1 nmol/ml biotinylated progastrin one hour at room temperature, and then stained bound peptides with anti-streptavidin-PE (Fisher Scientific). Streptavidin-PE alone staining was used as negative control.

### Analysis of apoptosis by TUNEL assay

The mice colonic tissues were deparaffinized and stained with In Situ Apoptosis Detection Kit (Chemicon) according to the manufacturer's instructions. The apoptotic index of TUNEL-positive cells was calculated using the total number of positive cells per field of sight at ten different locations in each colon under light microscopy.

### In situ hybridization assay

The mice colon frozen tissues were fixed with freshly prepared 4% paraformaldehyde (PFA) overnight, and immersed in 30% sucrose in PBS at 4°C until the tissue sinks to the bottom of the container. The colons were cut in 7μm sections, and the chromogenic detection of GPR56 was hybridized with the GPR56 mRNA oligo probes (Advanced Cell Diagnostics, Cat No. 318248) and In Situ Hybridization Detection Kit (Advanced Cell Diagnostics) according to the manufacturer's instructions, and the cells expressing GPR56 were analyzed under the light microscope.

### Statistics

Group measures are expressed as mean ± SD for all parameters determined, unless otherwise indicated. Statistical analysis was performed using 1-way ANOVA followed by a Tukey or Dunnet's test. *P* values less than 0.05 were considered statistically significant.

## Abbreviations

hGAS: Human progastrin
GPR56: G-protein coupled receptor 56
BrdU: 5-bromo-2’-deoxyuridine
BMP: Bone morphogenetic protein
